# Trends in and Characteristics of Drug Overdose Deaths Involving Illicitly Manufactured Fentanyls — United States, 2019–2020

**DOI:** 10.15585/mmwr.mm7050e3

**Published:** 2021-12-17

**Authors:** Julie O’Donnell, Lauren J. Tanz, R. Matt Gladden, Nicole L. Davis, Jessica Bitting

**Affiliations:** ^1^Division of Overdose Prevention, National Center for Injury Prevention and Control, CDC; ^2^National Network of Public Health Institutes, New Orleans, Louisiana.

During May 2020–April 2021, the estimated number of drug overdose deaths in the United States exceeded 100,000 over a 12-month period for the first time, with 64.0% of deaths involving synthetic opioids other than methadone (mainly illicitly manufactured fentanyls [IMFs], which include both fentanyl and illicit fentanyl analogs).* Introduced primarily as adulterants in or replacements for white powder heroin east of the Mississippi River (*1*), IMFs are now widespread in white powder heroin markets, increasingly pressed into counterfeit pills resembling oxycodone, alprazolam, or other prescription drugs, and are expanding into new markets, including in the western United States^†^ (*2*). This report describes trends in overdose deaths involving IMFs (IMF-involved deaths) during July 2019–December 2020 (29 states and the District of Columbia [DC]), and characteristics of IMF-involved deaths during 2020 (39 states and DC) using data from CDC’s State Unintentional Drug Overdose Reporting System (SUDORS). During July 2019–December 2020, IMF-involved deaths increased sharply in midwestern (33.1%), southern (64.7%), and western (93.9%) jurisdictions participating in SUDORS. Approximately four in 10 IMF-involved deaths also involved a stimulant. Highlighting the need for timely overdose response, 56.1% of decedents had no pulse when first responders arrived. Injection drug use was the most frequently reported individual route of drug use (24.5%), but evidence of snorting, smoking, or ingestion, but not injection drug use was found among 27.1% of decedents. Adapting and expanding overdose prevention, harm reduction, and response efforts is urgently needed to address the high potency (*3*), and various routes of use for IMFs. Enhanced treatment for substance use disorders is also needed to address the increased risk for overdose (*4*) and treatment complications (*5*) associated with using IMFs with stimulants.

Death certificate data, postmortem toxicology testing results, and death scene and witness findings from medical examiner or coroner reports are entered into SUDORS for unintentional drug overdose deaths and those of undetermined intent in 48 participating jurisdictions, providing comprehensive details about overdose deaths across jurisdictions not available from other data sources (*6*). IMFs^§^ were identified using toxicology and scene evidence (*7*). Monthly trends in IMF-involved deaths during July 1, 2019–December 31, 2020, were stratified by geographic region^¶^ among 30 jurisdictions with complete data (26 reported all overdose deaths in the jurisdiction and four reported deaths from subsets of counties).** Differences in the proportions of overdose deaths that involved IMFs (comparing July–December 2019 with July–December 2020) were assessed using z-tests, with p-values <0.05 considered statistically significant. Decedent demographics, overdose characteristics, and other drug co-involvement, were examined among 40 jurisdictions using 2020 data (35 reported all overdose deaths in the jurisdiction and five reported deaths from subsets of counties), stratified by region.^††^ Analyses were performed using SAS (version 9.4; SAS Institute). This activity was reviewed by CDC and conducted consistent with applicable federal law and CDC policy.^§§^

IMF-involved deaths increased from July–December 2019 to July–December 2020 across regions: Northeast (3.5% relative increase; from 5,019 to 5,194 deaths), Midwest (33.1%; 1,510 to 2,010), South (64.7%; 2,636 to 4,342), and West (93.9%; 955 to 1,852) (Figure 1). The proportions of drug overdose deaths involving IMFs increased significantly in midwestern (12.2% relative increase; from 62.9% to 70.6%), southern (24.1%; 54.3% to 67.4%), and western (45.7%; 30.2% to 44.0%) jurisdictions, while remaining stable in the Northeast (1.3% increase; 79.8% to 80.8%).

**FIGURE 1:**
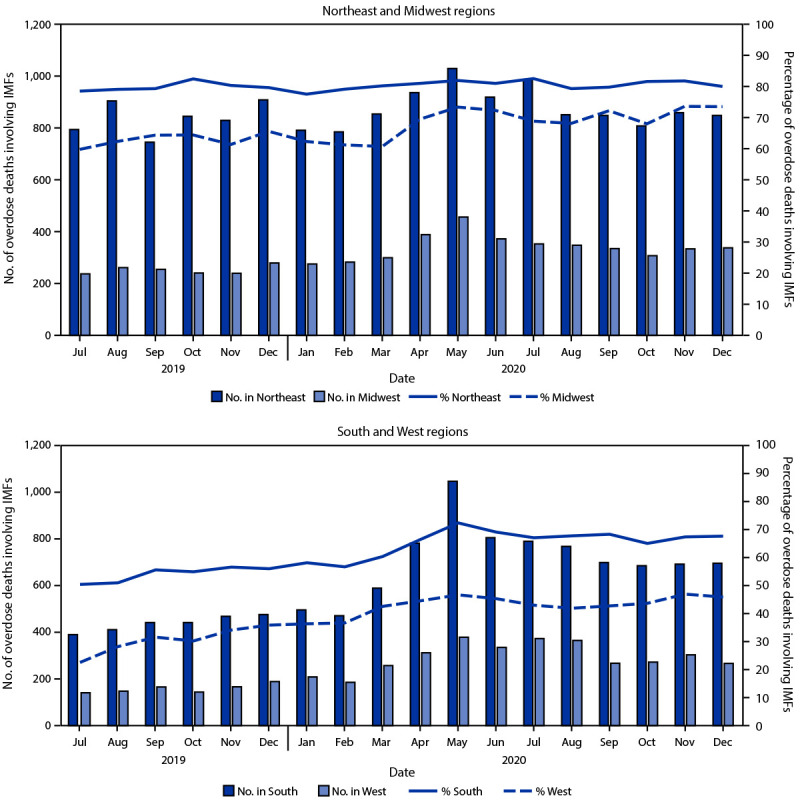
Number and percentage of drug overdose deaths involving illicitly manufactured fentanyls,* by month and geographic region^†^ — State Unintentional Drug Overdose Reporting System, 30 jurisdictions,^§^ July 2019–December 2020 **Abbreviations**: IMFs = illicitly manufactured fentanyls; SUDORS = State Unintentional Drug Overdose Reporting System. * Includes illicitly manufactured fentanyl and fentanyl analogs. ^†^ *Northeast:* Connecticut, Maine, Massachusetts, New Hampshire, New Jersey, Pennsylvania, Rhode Island, and Vermont; *Midwest:* Illinois, Kansas, Minnesota, Missouri, and South Dakota; *South:* Delaware, District of Columbia, Georgia, North Carolina, Oklahoma, Tennessee, Virginia, and West Virginia; *West:* Alaska, Arizona, Colorado, Montana, Nevada, New Mexico, Oregon, Utah, and Washington. ^§^ Illinois, Missouri, Pennsylvania, and Washington reported deaths from counties that accounted for ≥75% of drug overdose deaths in the state in 2017, per SUDORS funding requirements; all other jurisdictions reported deaths from the full jurisdiction.

Across regions, more IMF-involved deaths co-involved stimulants (40.1%–45.2%) than co-involved opioids other than IMFs (19.2%–31.6%) (Figure 2). Cocaine (25.5%–35.1%) and heroin (16.6%–22.3%) were the most commonly co-involved stimulant and opioid other than IMFs, respectively, among IMF-involved deaths in all regions except the West, where methamphetamine (25.3%) and prescription opioids (12.0%) were most common. Substantial proportions of IMF-involved deaths involved no other opioid or stimulant (Northeast: 39.7%; Midwest: 40.6%; South: 37.1%; West: 49.5%). Benzodiazepines, gabapentin, and xylazine, all nonopioids with sedative or hypnotic properties, were involved in 12.3%–15.5%, 2.7%–5.2%, and 0.1%–5.5% of IMF-involved deaths, respectively, across all regions.

**FIGURE 2:**
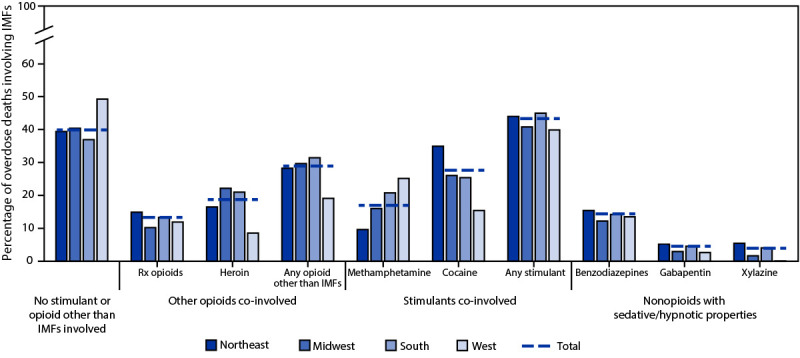
Co-involvement of other opioids, stimulants, and other psychoactive substances in drug overdose deaths involving illicitly manufactured fentanyls,* by geographic region^†^ — State Unintentional Drug Overdose Reporting System, 40 jurisdictions,^§^ 2020^¶^^,^**^,^^††^ **Abbreviations**: IMFs = illicitly manufactured fentanyls; Rx = prescription; SUDORS = State Unintentional Drug Overdose Reporting System. * Includes illicitly manufactured fentanyl and fentanyl analogs. ^†^ *Northeast:* Connecticut, Maine, Massachusetts, New Hampshire, New Jersey, Pennsylvania, Rhode Island, and Vermont; *Midwest:* Illinois, Iowa, Kansas, Michigan, Minnesota, Missouri, Nebraska, Ohio, and South Dakota; *South:* Arkansas, Delaware, District of Columbia, Georgia, Kentucky, Louisiana, Maryland, Mississippi, North Carolina, Oklahoma, Tennessee, Virginia, and West Virginia; *West:* Alaska, Arizona, Colorado, Hawaii, Montana, Nevada, New Mexico, Oregon, Utah, and Washington. ^§^ Illinois, Louisiana, Missouri, Pennsylvania, and Washington reported deaths from counties that accounted for ≥75% of drug overdose deaths in the state in 2017, per SUDORS funding requirements; all other jurisdictions reported deaths from the full jurisdiction. Jurisdictions included if data were available for January–June or July–December 2020, or both. ^¶^ Deaths in the “no stimulant or opioid other than IMFs involved” category could have involved drugs other than opioids and stimulants. The “any opioids other than IMFs” category includes heroin, prescription opioids, and other illicit synthetic opioids (e.g., isotonitazene, U-47700). The “any stimulant” category includes cocaine, amphetamines, cathinones, and other central nervous system stimulants (e.g., atomoxetine, caffeine). ** Buprenorphine and methadone are included as prescription opioids; however, they are used both for treatment of pain and for treatment of opioid use disorder. Fewer than 3% of deaths involved buprenorphine, and fewer than 4% of deaths involved methadone, across jurisdictions. ^††^ Co-involvement of gabapentin and xylazine in IMF deaths is likely underestimated because of lack of routine postmortem toxicology testing for these drugs across jurisdictions.

Most IMF-involved deaths (73.0%) were among males (Table). In western jurisdictions, 21.8% of decedents were aged <25 years, whereas in other regions, 5.9%–8.7% of decedents were in this age group. Injection drug use was the most commonly reported route of drug use among all IMF-involved deaths in all regions (22.7%–30.6%), except the West (11.7%). Evidence of snorting, smoking, or ingestion, but not injection drug use, was reported in 57.1% of deaths in western jurisdictions and 19.2%–26.4% of deaths in other regions. For 48.3% of IMF-involved deaths, no evidence of route of drug use was documented. Counterfeit pill evidence^¶¶^ was documented among 13.3% of deaths in the West and <1.0% in other regions. Approximately one half of decedents (56.1%) had no pulse when first responders arrived. Most deaths occurred at the decedent’s own home (64.2%) or in a house or apartment belonging to someone else (14.8%). Approximately one third (34.7%) of deaths occurred with a potential bystander*** present who did not respond to the overdose, most commonly because of spatial separation from the decedent (e.g., in a different room of the same house).

**TABLE:** Demographics and characteristics of drug overdose deaths involving illicitly manufactured fentanyls,* by geographic region^†^ — State Unintentional Drug Overdose Reporting System, 40 jurisdictions,^§^ 2020

Characteristic	No. (%)				
	Northeast	Midwest	South	West	Total
**Among all decedents**					
**Total**	**10,502**	**7,350**	**12,304**	**3,540**	**33,696**
**Gender^¶^**					
Male	7,872 (75.0)	5,303 (72.1)	8,816 (71.7)	2,609 (73.7)	**24,600 (73.0)**
Female	2,630 (25.0)	2,047 (27.9)	3,488 (28.3)	931 (26.3)	**9,096 (27.0)**
Unknown/Missing	0 (—)	0 (—)	0 (—)	0 (—)	**0 (—)**
**Age group, yrs^¶^**					
Median (IQR)	40 (32–51)	39 (31–51)	39 (31–50)	33 (26–43)	**39 (31–50)**
<15	—**	—**	13 (0.1)	21 (0.6)	**47 (0.1)**
15–24	623 (5.9)	636 (8.7)	971 (7.9)	750 (21.2)	**2,980 (8.8)**
25–34	2,791 (26.6)	2,052 (27.9)	3,474 (28.2)	1,110 (31.4)	**9,427 (28.0)**
35–44	2,914 (27.8)	1,863 (25.4)	3,379 (27.5)	833 (23.5)	**8,989 (26.7)**
45–54	2,210 (21.0)	1,474 (20.1)	2,396 (19.5)	470 (13.3)	**6,550 (19.4)**
55–64	1,620 (15.4)	1,065 (14.5)	1,724 (14.0)	305 (8.6)	**4,714 (14.0)**
≥65	338 (3.2)	250 (3.4)	345 (2.8)	50 (1.4)	**983 (2.9)**
Unknown/Missing	—**	—**	—**	—**	—**
**Race/Ethnicity^¶^**					
White, non-Hispanic	7,297 (70.4)	4,599 (62.9)	8,444 (69.2)	1,905 (54.3)	**22,245 (66.6)**
Black, non-Hispanic	1,622 (15.6)	2,010 (27.5)	3,072 (25.2)	289 (8.2)	**6,993 (20.9)**
AI/AN, non-Hispanic	23 (0.2)	92 (1.3)	103 (0.8)	154 (4.4)	**372 (1.1)**
A/OPI, non-Hispanic	65 (0.6)	38 (0.5)	60 (0.5)	45 (1.3)	**208 (0.6)**
Multiple races, non-Hispanic	49 (0.5)	63 (0.9)	71 (0.6)	60 (1.7)	**243 (0.7)**
Hispanic	1,316 (12.7)	504 (6.9)	455 (3.7)	1,054 (30.1)	**3,329 (10.0)**
Unknown/Missing	130	44	99	33	**306**
**Among decedents with data from coroner or medical examiner reports**					
**Total**	**9,840**	**7,067**	**10,959**	**3,505**	**31,371**
**Drug use history^††^**					
Illicit opioids	2,746 (27.9)	2,689 (38.1)	3,695 (33.7)	915 (26.1)	**10,045 (32.0)**
Prescription opioids	462 (4.7)	522 (7.4)	889 (8.1)	832 (23.7)	**2,705 (8.6)**
Unspecified opioids	657 (6.7)	389 (5.5)	473 (4.3)	223 (6.4)	**1,742 (5.6)**
Cocaine	923 (9.4)	804 (11.4)	1,282 (11.7)	355 (10.1)	**3,364 (10.7)**
Methamphetamine	229 (2.3)	392 (5.5)	572 (5.2)	441 (12.6)	**1,634 (5.2)**
Other	3,684 (37.4)	2,222 (31.4)	3,916 (35.7)	1,150 (32.8)	**10,972 (35.0)**
**Route of drug use** ^§§^					
Injection	2,238 (22.7)	1,691 (23.9)	3,353 (30.6)	411 (11.7)	**7,693 (24.5)**
No injection reported; snorting, smoking, or ingestion reported	1,887 (19.2)	1,865 (26.4)	2,756 (25.1)	2,002 (57.1)	**8,510 (27.1)**
No injection; snorting	1,017 (10.3)	931 (13.2)	1,520 (13.9)	835 (23.8)	**4,303 (13.7)**
No injection; smoking	628 (6.4)	628 (8.9)	962 (8.8)	987 (28.2)	**3,205 (10.2)**
No injection; ingestion	467 (4.7)	774 (11.0)	914 (8.3)	1,012 (28.9)	**3,167 (10.1)**
No reported route of drug use	5,708 (58.0)	3,507 (49.6)	4,841 (44.2)	1,087 (31.0)	**15,143 (48.3)**
**Evidence of counterfeit pills**	23 (0.2)	63 (0.9)	46 (0.4)	466 (13.3)	**598 (1.9)**
**Documentation of no pulse at first responder arrival^¶^**	4,789 (48.8)	2,832 (40.3)	7,410 (69.4)	2,354 (67.8)	**17,385 (56.1)**
**Potential bystander present^¶^**	4,262 (43.3)	2,931 (41.5)	6,053 (55.2)	2,234 (63.7)	**15,480 (49.3)**
**Potential bystander present but no documented overdose response efforts** ^¶¶^	2,860 (29.1)	2,271 (32.1)	4,212 (38.4)	1,528 (43.6)	**10,871 (34.7)**
Did not recognize abnormalities	230 (8.0)	288 (12.7)	293 (7.0)	209 (13.7)	**1,020 (9.4)**
Recognized abnormalities but not as overdose	226 (7.9)	205 (9.0)	361 (8.6)	207 (13.5)	**999 (9.2)**
Bystander also using drugs or drinking	223 (7.8)	284 (12.5)	416 (9.9)	110 (7.2)	**1,033 (9.5)**
Spatial separation	1,189 (41.6)	1,055 (46.5)	1,860 (44.2)	901 (59.0)	**5,005 (46.0)**
Unaware decedent was using drugs	231 (8.1)	370 (16.3)	635 (15.1)	330 (21.6)	**1,566 (14.4)**
**Drug use witnessed^¶^**	644 (6.5)	612 (8.7)	1,142 (10.4)	463 (13.2)	**2,861 (9.1)**
**Overdose at home^¶^**	6,267 (66.2)	4,249 (62.6)	6,068 (61.9)	2,348 (68.3)	**18,932 (64.2)**
**Overdose in house or apartment; not own home^¶^**	1,200 (12.8)	1,134 (16.7)	1,610 (16.3)	409 (12.0)	**4,353 (14.8)**

**Abbreviations**: AI/AN = American Indian or Alaska Native; A/OPI = Asian or Other Pacific Islander; IMFs = illicitly manufactured fentanyls; SUDORS = State Unintentional Drug Overdose Reporting System.

* Includes illicitly manufactured fentanyl and fentanyl analogs.

^†^ *Northeast:* Connecticut, Maine, Massachusetts, New Hampshire, New Jersey, Pennsylvania, Rhode Island, and Vermont; *Midwest:* Illinois, Iowa, Kansas, Michigan, Minnesota, Missouri, Nebraska, Ohio, and South Dakota; *South:* Arkansas, Delaware, District of Columbia, Georgia, Kentucky, Louisiana, Maryland, Mississippi, North Carolina, Oklahoma, Tennessee, Virginia, and West Virginia; *West:* Alaska, Arizona, Colorado, Hawaii, Montana, Nevada, New Mexico, Oregon, Utah, and Washington.

^§^ Illinois, Louisiana, Missouri, Pennsylvania, and Washington reported deaths from counties that accounted for ≥75% of drug overdose deaths in the state in 2017, per SUDORS funding requirements; all other jurisdictions reported deaths from the full jurisdiction. Jurisdictions included if data were available for January–June or July–December 2020, or both. Data for July–December 2020 for one state (816 deaths) only included in section for all decedents, because the overall percentage of decedents with a medical examiner or coroner report was <75%, which is the cut-off used in SUDORS for inclusion in analyses of overdose circumstances.

^¶^ Missing values were excluded from calculations of percentages. Percentages might not sum to 100% because of rounding.

** Data suppressed because cell contained <10 deaths or to prevent calculation of another suppressed cell.

^††^ Drug use history categories are not mutually exclusive; a decedent could have a documented history of use or misuse of more than one type of drug. Illicit opioid use history includes history of use of IMFs or heroin. Other drug use history includes history of benzodiazepine misuse, history of cannabis use, history of unspecified drug use, and other drug use history (with specific drugs written in).

^§§^ Route of drug use cannot be directly linked to specific drugs if more than one drug detected and more than one route reported (e.g., if there was evidence of injection and snorting, both would be documented; if more than one drug was detected, it cannot be determined which was injected and which was snorted). Percentages for all rows in this section calculated out of the region total. Categories for no injection/snorting, no injection/smoking, and no injection/ingestion are not mutually exclusive; a death could have evidence of more than one of these routes. Other routes of drug use (transdermal, suppository, sublingual, buccal) were each reported for <0.5% of deaths in each region so these routes were not included but account for why the totals for “injection,” “no injection reported; snorting, smoking, or ingestion reported,” and “no reported route of drug use” do not sum to the regional totals.

^¶¶^ Reasons for lack of bystander response are presented with percentages calculated out of deaths with evidence of a potential bystander present, but with no evidence that any bystander response was made (e.g., no naloxone administered and no cardiopulmonary resuscitation performed). Reasons for no response are not mutually exclusive; more than one reason could be reported per death.

## Discussion

This report highlights four main findings regarding IMF-involved deaths: 1) deaths increased sharply in midwestern, southern, and western jurisdictions during 2019–2020; 2) approximately four in 10 deaths also involved stimulants; 3) approximately one half of decedents had no pulse when first responders arrived; and 4) evidence of injection was the most frequently documented route of drug use, but substantial percentages of deaths likely involved other routes, especially in the West. Rapid increases in IMF-involved deaths during 2019–2020, which accelerated during the COVID-19 pandemic,^†††^ suggest increases in IMF distribution and exposure, consistent with law enforcement drug supply data (*8*), with evidence of plateauing of IMF-involved deaths only in the Northeast. Lower but increasing percentages of IMF-involved overdoses in southern and western jurisdictions, versus high percentages in northeastern and midwestern jurisdictions, and increases in IMF supply during 2020 (*8*) raise concerns about the potential for continued increases in IMF-involved deaths in jurisdictions in these regions.

Substantial stimulant co-involvement in IMF-involved deaths reflects recent trends in concurrent IMF and stimulant use (*4*,*6*), which can complicate substance use disorder treatment (*5*) and increase overdose risk (*4*). IMF-involved deaths involving any stimulant and those involving no other opioids or stimulants were more common than were those involving another opioid, suggesting that IMFs are well-established in many drug markets, independent of heroin. Co-involvement of benzodiazepines, gabapentin, and xylazine in some IMF-involved deaths is particularly dangerous because their sedative or hypnotic properties do not respond to naloxone.^§§§^ Overdose response messaging must emphasize calling 9-1-1 and seeking further treatment, even after naloxone administration.

A challenge in responding to IMF overdoses is that approximately one half of decedents had no pulse when first responders arrived, reducing their chance of survival. This statistic highlights both the high potency of IMFs (*3*) and the potential for rapid overdose^¶¶¶^ and underscores the need to enhance harm reduction efforts, including improving naloxone access and distribution for persons who use drugs (and their family members and friends) to ensure timely response to IMF overdoses. In the approximately one third of deaths where potential bystanders provided no response, common barriers were spatial separation, lack of awareness of drug use, and inability to recognize a drug overdose. Thus, expanding education about drug use signs and overdose recognition and response, and the importance of regularly checking on family or friends who potentially use drugs, might reduce mortality. Efforts to reduce fatal overdoses at home (e.g., encouragement of testing drug products with fentanyl test strips, having naloxone available, and avoidance of using drugs alone) are needed, because most IMF-involved deaths occurred in the decedent’s own home.

Although injection was the most commonly reported route of drug use among IMF-involved deaths, in approximately one quarter of deaths, including approximately one half of deaths in western jurisdictions, there was evidence of snorting, smoking, or ingestion, but not injection. A September 2021 Drug Enforcement Administration public safety alert described rapid increases in counterfeit pill availability and variety,**** and this might help explain the relatively high percentage of decedents with no documented injection drug use. Evidence of counterfeit pills (which can be ingested orally or prepared for snorting, injecting, or smoking) was found in <1% of IMF-involved deaths overall but in 13.3% of IMF-involved deaths in western jurisdictions. This statistic is, however, likely a significant underestimation of counterfeit pill involvement because identification of pills as counterfeit on the basis of appearance can be difficult, and testing of pills found at the scene is rarely done; documenting counterfeit pill evidence is therefore challenging. IMF availability in pill form is likely contributing to its increased use across the United States, especially in western drug markets where white powder heroin is uncommon (*9*). Coupled with local reports,^††††^ the finding of counterfeit pill evidence in IMF-involved deaths highlights the need for enhanced surveillance for overdoses involving counterfeit pills and education about counterfeit pills containing IMFs, as persons might be unaware that they contain IMFs or even opioids (e.g., if using counterfeit pills designed to look like nonopioid medications such as alprazolam). One western city reported a shift from injecting opioids to smoking IMFs (*9*); however, the extent to which this shift is occurring elsewhere is unknown. Investigating the higher proportion of IMF-involved deaths among young persons in the West and whether and how these deaths are linked to counterfeit pills and other routes of use is needed. Persons using IMFs by routes other than injection might not use traditional harm reduction services such as syringe services programs, or might be newer to drug use (*10*), and therefore might be harder to reach than persons injecting drugs. Expanding the focus of interventions within and beyond such traditional avenues to reach persons using IMFs by other routes, while enhancing existing efforts to address risks associated with injecting IMFs, could help prevent overdoses.

The findings in this report are subject to at least three limitations. First, the jurisdictions included (30 in trend analyses and 40 in descriptive analyses) are not nationally representative, and some jurisdictions report data from subsets of counties; therefore, these findings might not be able to be extrapolated to other areas. Second, death investigation differs across and within jurisdictions and might contribute to regional differences. Also, difficulties in obtaining overdose characteristic evidence for some deaths (e.g., those with no witnesses) can lead to underestimation (e.g., drug use route was unknown for approximately one half of deaths). Finally, there is no standard for postmortem toxicology testing or drug involvement determination, potentially resulting in failure to detect IMFs or other drugs.

Urgent action is needed to slow and reverse rapid increases in drug overdose deaths involving IMFs and other drugs, including enhancing access to substance use disorder treatment (e.g., medications for opioid use disorder) and expanding harm reduction approaches that address risk factors associated with IMFs (e.g., improving and expanding distribution of naloxone to persons who use drugs and their friends and family,^§§§§^ distributing fentanyl test strips to test drug products for fentanyl, and increasing overdose education and access to comprehensive syringe services programs). Innovative approaches are needed to address the endemic nature of IMF-involved overdoses, noninjection routes of IMF use, and frequent polysubstance use, in particular, the rising use of opioids and stimulants.

**Summary**

**What is already known about this topic?**

Synthetic opioids, including illicitly manufactured fentanyls (IMFs), were involved in 64% of >100,000 estimated U.S. drug overdose deaths during May 2020–April 2021.

**What is added by this report?**

During 2019–2020, IMF-involved overdose deaths increased sharply in midwestern, southern, and western jurisdictions. During 2020, approximately 40% of IMF-involved deaths also involved stimulants, and 56% of decedents had no pulse when first responders arrived. Injection drug use was reported in 25% of deaths, and noninjection routes of drug use in 27% of deaths.

**What are the implications for public health practice?**

Adapting overdose prevention and response efforts to address risk factors associated with IMFs and using innovative approaches to address the endemic nature of IMFs, various routes of IMF use, and frequent polysubstance use could slow increases in IMF-involved deaths.

## Footnotes

* https://www.cdc.gov/nchs/nvss/vsrr/drug-overdose-data.htm (Accessed November 29, 2021).

†
https://www.dea.gov/sites/default/files/2021-02/DIR-008-21%202020%20National%20Drug%20Threat%20Assessment_WEB.pdf

§ Fentanyl was classified as likely illicitly manufactured using toxicology, scene, and witness evidence. In the absence of sufficient evidence to classify fentanyl as illicit or prescription (<12% of deaths involving fentanyl), fentanyl was classified as illicit because the vast majority of fentanyl overdose deaths involve illicit fentanyl. All fentanyl analogs except alfentanil, remifentanil, and sufentanil (which have legitimate human medical use) were included as illicitly manufactured fentanyls.

¶ U.S. Census regions were used to stratify jurisdictions into geographic regions: https://www2.census.gov/geo/pdfs/maps-data/maps/reference/us_regdiv.pdf. Not all jurisdictions in each region are included in analyses: trend analyses include eight of nine jurisdictions in the Northeast region, five of 12 jurisdictions in the Midwest region, eight of 17 jurisdictions in the South region, and nine of 13 jurisdictions in the West region; analyses of overdose characteristics include eight of nine jurisdictions in the Northeast region, nine of 12 jurisdictions in the Midwest region, 13 of 17 jurisdictions in the South region, and 10 of 13 jurisdictions in the West region.

** Jurisdictions included: Alaska, Arizona, Colorado, Connecticut, Delaware, District of Columbia, Georgia, Illinois, Kansas, Maine, Massachusetts, Minnesota, Missouri, Montana, Nevada, New Hampshire, New Jersey, New Mexico, North Carolina, Oklahoma, Oregon, Pennsylvania, Rhode Island, South Dakota, Tennessee, Utah, Vermont, Virginia, Washington, and West Virginia. Illinois, Missouri, Pennsylvania, and Washington reported deaths from counties that accounted for ≥75% of drug overdose deaths in the state in 2017, per SUDORS funding requirements; all other jurisdictions reported deaths from the full jurisdiction.

†† Jurisdictions included: Alaska, Arizona, Arkansas, Colorado, Connecticut, Delaware, District of Columbia, Georgia, Hawaii, Illinois, Iowa, Kansas, Kentucky, Louisiana, Maine, Maryland, Massachusetts, Michigan, Minnesota, Mississippi, Missouri, Montana, Nebraska, Nevada, New Hampshire, New Jersey, New Mexico, North Carolina, Ohio, Oklahoma, Oregon, Pennsylvania, Rhode Island, South Dakota, Tennessee, Utah, Vermont, Virginia, Washington, and West Virginia. Illinois, Louisiana, Missouri, Pennsylvania, and Washington reported deaths from counties that accounted for ≥75% of drug overdose deaths in the state in 2017, per SUDORS funding requirements; all other jurisdictions reported deaths from the full jurisdiction. Jurisdictions were included if data were available for January–June, July–December 2020, or both.

§§ 45 C.F.R. part 46.102(l)(2), 21 C.F.R. part 56; 42 U.S.C. Sect. 241(d); 5 U.S.C. Sect. 552a; 44 U.S.C. Sect. 3501 et seq.

¶¶ Counterfeit pill evidence included evidence that potential counterfeit pills were found at the scene of the fatal overdose or were consumed by the decedent (according to witness report). Evidence consistent with counterfeit pills included unmarked pills; pills marked with M30 or otherwise appearing like oxycodone pills, with no oxycodone detected by postmortem toxicology testing; pills appearing like alprazolam pills, with no alprazolam detected; pills noted to be counterfeit or potentially counterfeit in the medical examiner or coroner report; and pills noted to have contained fentanyl or tested positive for fentanyl. Detail about potential counterfeit pills in medical examiner or coroner reports varies widely, and some evidence was likely included in error and some evidence missed. It is also possible that counterfeit pills were on scene but not consumed by the decedent.

*** For SUDORS, a potential bystander is defined as a person aged ≥11 years who was physically nearby either during or shortly preceding a drug overdose and potentially had an opportunity to intervene or respond to the overdose. This includes any persons in the same structure (e.g., same room or same building, but different room) as the decedent during that time. For example, the family member of an opioid overdose decedent who was in another room during the fatal incident would be considered a potential bystander if that person might have had an opportunity to provide life-saving measures such as naloxone administration, if adequate resources were available and the family member was aware that an overdose event could occur. This does not include, however, persons in different self-contained parts of larger buildings (e.g., a person in a different apartment in the same apartment building would not be considered a potential bystander).

†††
https://emergency.cdc.gov/han/2020/han00438.asp

§§§
https://www.cdc.gov/mmwr/volumes/70/wr/mm7034a4.htm

¶¶¶
https://www.cdc.gov/mmwr/volumes/66/wr/mm6614a2.htm

****
https://www.dea.gov/onepill

†††† Additional information is available from local reports: San Diego, California (https://www.dea.gov/press-releases/2020/08/06/alarming-spike-fentanyl-related-overdose-deaths-leads-officials-issue); Multnomah County, Oregon (https://www.multco.us/multnomah-county/news/health-officials-warn-rise-deaths-counterfeit-pills); Anchorage, Alaska (https://local.nixle.com/alert/7943584/?sub_id); King County, Washington (https://publichealthinsider.com/2021/08/17/thirty-four-king-county-residents-died-from-fentanyl-drug-overdose-in-july-how-our-community-can-take-action/).

§§§§ September 2021 Model Expanded Access to Emergency Opioid Antagonists Act: https://legislativeanalysis.org/model-expanded-access-to-emergency-opioid-antagonists-act/
